# Effect of a Reduced-Protein Diet Supplemented with Essential Amino Acids on the Muscle Proteome of Female and Entire Male Finishing Pigs

**DOI:** 10.3390/ani15223325

**Published:** 2025-11-18

**Authors:** André M. de Almeida, Hugo Osório, María Ángeles Latorre, Javier Álvarez-Rodríguez

**Affiliations:** 1 LEAF—Linking Landscape, Environment, Agriculture and Food Research Center, Associated Laboratory TERRA, Instituto Superior de Agronomia, Universidade de Lisboa, Tapada da Ajuda, 1349-017 Lisboa, Portugal; 2 i3S—Instituto de Investigação e Inovação em Saúde, Universidade do Porto, 4200-135 Porto, Portugal; hosorio@i3s.up.pt; 3 IPATIMUP—Institute of Molecular Pathology and Immunology of the University of Porto, Universidade do Porto, 4200-135 Porto, Portugal; 4 Departamento de Patologia, Faculdade de Medicina, Universidade do Porto, 4200-319 Porto, Portugal; 5 Departamento de Producción Animal y Ciencia de los Alimentos, Facultad de Veterinaria, Universidad de Zaragoza-IA2, Calle Miguel Servet 177, 50013 Zaragoza, Spain; malatorr@unizar.es; 6 Departamento de Producción Animal y Ciencia de los Alimentos, Escuela Politécnica Superior, Universidad de Zaragoza-IA2, Carretera de Cuarte s/n, 22071 Huesca, Spain

**Keywords:** amino acid supplementation, proteome, reduced protein content, sex

## Abstract

Crude protein (CP) decrease coupled to amino acid (AAs) supplementation is interesting for swine nutrition. A proteomics approach unraveled physiological events underlying differences between sexes fed two diets: control and low CP in fast-growing Duroc X (Landrace X Large white) finishing pigs. Sixty animals per sex were distributed in pens (five replicate pens/group), fed *ad libitum* with two isoenergetic diets (145 vs. 125 g CP/kg with AA supplementation; control and low-CP) for 42 days. Semimembranosus muscle was sampled. Five samples per group were used for Label Free proteomics. Four comparisons were considered: Control Males vs. Control Females; Control Females vs. Low CP females; Control males vs. Low CP males, and Low CP males vs. Low CP females, identifying, respectively, 26, 19, 12, and 11 DAPs (Differentially Accumulated Proteins). Control males had higher abundance of proteins related to cell differentiation and growth compared to females, highlighting continual muscle accretion in the former and lipogenesis onset in the latter. Control females and males had increased DAPs related to tissue growth and differentiation compared to Low CP animals that had increased lipid accretion. Both sexes reacted similarly to the two diets. Low CP diet led to advanced lipogenesis and decreased muscle accretion pathways.

## Simple Summary

Dietary Crude protein (CP) reduction with amino acid (AA) supplementation pig feeding is an interesting cost-reducing strategy with benefits to the environment. Physiological studies addressing fast-growing Duroc sire line finishing pigs of both sexes are non-existent. We hypothesized that entire males and females will be similarly affected by such a strategy. We conducted a study with 60 male and 60 female finishing pigs subjected to two isoenergetic diets differing in CP content (145 and 125 g CP/kg with AA supplementation, control, and low-CP). We established proteomics profiles of the Semimembranosus muscle. Males and females reacted alike to the two diets, although low CP diet led to advanced lipogenesis and decreased muscle growth. The strategy can be used in fast-growing Duroc sire line genotypes of both sexes in the finishing phase, albeit that implies a slight modification of pathways related to muscle protein and lipid synthesis without compromising productive performance.

## 1. Introduction

One of the most efficient ways of reducing costs and environmental negative externalities associated with pig production is lowering Crude Protein (CP) contents in feeds, coupling it to essential amino acid (AA) supplementation. Pig production has considerable environmental impacts, namely through international commerce of feedstuffs such as corn and particularly soybean meal that has to be imported to Europe from North and South America. Furthermore, the use of high levels of CP in feeds often leads to increased emissions, with special relevance to NH3, associated with animal excreta in effluent and its storage [1]. The reduction of CP contents in swine feeds is possible because dietary AA requirements can be complemented without excessive dietary CP by increasing crystalline essential AA incorporation [1]. This is a very efficient strategy, as demonstrated in piglets [2], growers, and finishers [3,4] of different genetic lines. Furthermore, it is often used in pork production systems worldwide. For further details on the strategy, kindly refer to the recent review by Almeida et al. [1]. Results vary considerably, with animals fed below CP recommendations and supplemented with crystalline AA having similar growth performances, carcass, and meat traits to those of control animals [1]. Furthermore, there are environmental benefits that include ammonia and Greenhouse gas (GHG) emissions in pig slurry production and storage with reduced acidification, eutrophication, and odor emissions [5]. The strategy is, however, very dependent on genetic type and sex, mostly due to divergent lean growth requirements, as well as endocrine differences around puberty between entire males, castrates, and females [1].

Modern pork production, such as that conducted in Europe, with particular relevance to Spain and Portugal, generally uses lean genotypes based on Pietrain X (Landrace X Large white) crosses. Due to their lean meat, such genotypes are unsuitable for the production of traditional high-value premium fresh pork products. Indeed, for such products, the pork industry often uses Duroc sires, crossed either with Iberian breeds [6] or with Landrace X Large white commercial dam lines [7]. Such animals are grown until heavier weights (120–150 kg) than lean Pietrain-sired genotypes (100 kg). Dietary CP reduction coupled to crystalline AA supplementation has seldom been described in heavier genotypes [1], particularly fast-growing animals, typically using Duroc Sires and Landrace X Large white sows. These are employed to target an upscale market in order to improve organoleptic traits of meat without penalties on feed conversion rate and thereby economic revenues. Furthermore, the majority of the studies on dietary CP reduction concern young animals, namely piglets or growers, with very few studies addressing finishing animals or comparison between the two sexes [1]. This is, however, very important as nutritional recommendations for growers and finishers are the same for both sexes that have nonetheless different nutritional needs and would thus be differently affected by this strategy [8]. We have recently conducted a study with male and female finishing pigs fed two isoenergetic diets with different CP contents (145 g CP/kg versus 125 g CP/kg with crystalline AA supplementation) [9]. We have demonstrated that males had similar performances when fed the two diets, albeit with higher weights and average daily gains than females. Both sexes had, furthermore, analogous carcass and meat traits, likely implying that females are slightly more precocious than entire males. Finally, control animals showed lower fat and saturated fat contents than pigs under the reduced CP diets. We have, thus, proven the usefulness of the strategy in fast-growing Duroc sired finishers of both sexes.

Over the last 2 decades, proteomics tools have been widely used in animal and veterinary sciences for a variety of purposes in different species [10,11]. These include meat science [12,13] in topics such as meat tenderization [14], sex [15], and breed differences [16] or the effect of different diets [17]. Indeed, and despite the difficulties inherent to misrepresentation of some domestic species in public databases, in addition to other limitations [18], proteomics has proven to be a very valuable tool, able to comprehensively describe the molecular changes underlying the studied factors. The use of proteomics is particularly suitable in the pig (*Sus scrofa*) species as protein databases for this animal have long been established [19], leading to studies that thoroughly describe changes occurring for instance in muscle tissue of pigs fed diets with the incorporation for instance of algae or microalgae [20,21].

The objective of this work is to study in male and female finishing pigs, the physiological effects at the muscle level inherent to a 2% CP reduction with crystalline AA supplementation using a label free proteomics approach. We hypothesized that the two sexes will have a different physiological response highlighting important contrasts regarding the metabolic pathways being upregulated.

## 2. Materials and Methods

### 2.1. Animals, Experimental Design and Muscle Sampling

Details regarding animals and experimental design have been previously described [9]. Briefly, 120 Duroc × (Landrace × Large White), 60 females and 60 entire males, were used in this experiment. Animals were obtained at 9 weeks of age and divided by split-sex pens (six pigs per pen). At 15 weeks of age (67.9 ± 1.75 kg), the experiment started when animals were fed *ad libitum* with one of two experimental diets for the last 6 weeks of the finishing phase. Per diet, 30 entire males and 30 females were used. The two experimental diets differed in CP content (145 versus 125 g CP/kg, respectively, Control CP and Low CP). Both diets were formulated in order to comply with the ideal protein concept under the FEDNA nutrient recommendations [8]. The low-CP diet had a reduction in protein sources and crystalline AAs were added in order to avoid essential AA deficiencies. Both diets were isoenergetic (10.2 MJ Net Energy/kg), had a similar electrolyte balance, and were fed in the pelleted form. Five pens per diet and sex were used. Feed composition is detailed in Supplementary Material S1 and highlights differences in CP and amino acids inclusion. For further details, please refer to the previous description [9]. Water and feed were provided *ad libitum*. After 42 days of the experimental period, animals were slaughtered in a commercial abattoir following standard commercial procedures. The study abided by the guidelines of the Spanish Animal Protection Regulations RD 53/2013, complying with European Union Directive 2010/63. Kindly refer to our previous manuscript for further details [9]. Upon slaughter, Semimembranosus muscle medial slices were excised from the half carcass (after 3 h of a supercooling period). In such excision, approximately 1–1.5 g were sampled from the same muscle site, using a scalpel. Samples were placed in a 2 mL microcentrifuge tube and immediately snap-frozen in liquid Nitrogen and kept at −80 °C until further analysis.

### 2.2. Protein Extraction

Protein extraction was conducted as detailed [22]. Briefly, five randomly chosen frozen samples from each of the four groups (Male control, Female control, Male Low CP, and Female Low CP) were processed individually in 2 mL microcentrifuge tubes. Each tube contained lysing matrix A (MP Biomedicals, Irvine, CA, USA) and a lysis buffer (100 mM of Tris-HCl, pH 8.5, 1% sodium deoxycholate, 10 mM of tris (2-carboxyethyl) phosphine (TCEP), 40 mM of chloroacetamide (CAA), and proteinase inhibitors). Protein extracts were homogenized using FastPrep-24 equipment (MP Biomedicals, Irvine, CA, USA) at 6.0 m/s, in three cycles of 30 s each, with 5 min intervals on ice. Subsequently, extracts were centrifuged for 5 min at 13,400 rpm using a benchtop microcentrifuge and transferred to 1.5 mL of low protein-binding tubes. Subsequently, Extracts were then incubated at 95 °C at 1000 rpm in a Thermomixer (Eppendorf, Hamburg, Germany) for 10 min and sonicated for 10 cycles, with 30 s on and 30 s off at 4 °C (Bioruptor, Diagenode, Liège, Belgium), which were then re-centrifuged. The obtained clarified lysates were transferred to new 1.5 mL tubes to ascertain protein concentration. A total of 100 micrograms of protein per sample was used for proteomics analysis using the solid-phase sample preparation (SP3) protocol [23]. Enzymatic digestion was conducted using trypsin/LysC (2 µg) for 16 h at 37 °C and 1000 rpm. Peptide concentration was determined by fluorescence.

### 2.3. Proteomics Data Acquisition

Protein identification and quantitation were conducted using nanoLC-MS/MS through a Field Asymmetric Ion Mobility Spectrometry—FAIMS interface. The instrument has a Vanquish Neo liquid chromatography system together with an Eclipse Tribrid Quadrupole, Orbitrap, Ion Trap mass spectrometer (Thermo Scientific, San Jose, CA, USA). A total of 250 nanograms of peptides per sample were loaded onto a trapping cartridge (PepMap Neo C18, 300 μm × 5 mm i.d., 174500, Thermo Scientific, Bremen, Germany). Subsequently, the trap column was in-line connected to an Aurora Frontier XT 60 cm, 75 μm (AUR4-60075C18-XT) chromatographic separation column (Ionopticks, Collingwood, VIC, Australia). A 116 min separation was accomplished by mixing A: 0.1% FA and B: 100% ACN, 0.1% FA with a flow gradient of 250 nL/min: 2 min (0% B to 4% B), 20 min (4% B to 12% B), 65 min (12% B to 28% B), 11 min (28% B to 45% B), 2 min (45% B to 85% B) and 16 min at 99% B. Afterwards, the column was equilibrated with 0% B. Data acquisition was conducted with Xcalibur 4.6 and Tune 4.0.4091 software (Thermo Scientific, Bremen, Germany).

MS results were acquired using the Data Dependent Acquisition—DDA procedure. MS acquisition was achieved with the Orbitrap detector at 120,000 resolution in positive mode, quadrupole isolation, scan range (*m*/*z*) 375–1500, RF Lens 30%, standard AGC target, and maximum injection time was set on auto, 1 microscan, data type profile, and without source fragmentation. FAIMS mode: standard resolution; total carrier gas flow: static 4 L/min, FAIMS CV: −45, −60, and −75 (cycle time, 1 s). Internal Mass calibration: Run-Start Easy-IC. Filters: MIPS; monoisotopic peak determination: peptide; charge state: 2–7, dynamic exclusion 30 s, intensity threshold, 5 × 10^3^. MS/MS data acquisition parameters: quadrupole isolation window 1.8 (*m*/*z*); activation type: HCD (30% CE); detector: ion trap; IT scan rate: rapid; mass range: normal; scan range mode: auto, normalized AGC target 100%; maximum injection time: 35 ms, data-type centroid.

Raw data were treated using Proteome Discoverer 3.1.1.93 (Thermo Scientific, San Jose, CA, USA) and searched against the UniProt database for the pig (*Sus scrofa*) proteome (2024_04 with 46,174 entries). Furthermore, the standard protein contaminant list (MaxQuant) was included in the analysis procedure. Sequest HT search engine was employed to identify tryptic peptides. Ion mass tolerance was set as 10 ppm for precursor ions and 0.5 Da for fragment ions. The maximum allowed missed cleavage sites were two. Cysteine carbamidomethylation was defined as constant modification. Methionine oxidation, deamidation of glutamine and asparagine, peptide terminus glutamine to pyroglutamate, and protein N-terminus acetylation, Met-loss, and Met-loss + acetyl were defined as the variable modifications. Peptide confidence was set to high. The processing node Percolator was enabled with the ensuing settings: maximum delta Cn 0.05; target FDR (strict) was set to 0.01, and target FDR (relaxed) was set to 0.05, validation based on q-value. Protein label-free quantitation was performed using the Minora feature detector node at the processing step. Precursor ions quantification was achieved at the consensus step with the parameters: inclusion of unique plus razor peptides, precursor abundance based on intensity, and normalization based on total peptide amount. For hypothesis testing, protein ratio calculation was pairwise ratio-based, and a t-test (background based) hypothesis test was performed.

### 2.4. Data Analysis

Samples were grouped and compared with each other, removing proteins with more than 50% missing values per experimental group. Protein filters of at least two peptides and one unique peptide were applied. The following criteria were used: fold change (FC) > 2 for upregulated proteins and FC < 0.5 for downregulated proteins. Protein accumulations were considered significantly different in each comparison when *p* < 0.05. RStudio environment on R software (version 4.2.2) was used to plot the principal component analysis (PCA), using packages FactoMineR and factoextra as described [24].

## 3. Results

### 3.1. Growth Performance and Meat Traits

Growth performance and meat quality traits have been previously described and are herein referenced for contextual purposes only. Kindly refer to Almeida et al. [18] for further details. Briefly, males fed the two diets were 7.25% heavier (*p* < 0.001), reaching a final body weight of 119.6–121.6 kg, whereas females had final body weights ranging from 111.6–113.3 kg. Similarly, significant differences were also recorded between males and females of both treatments (*p* < 0.0001) concerning average daily gains. These were higher in the former (1.23 vs. 1.01 kg/day). As expected, the feed conversion ratio was lower for males of both treatments (2.12–2.28) than for females also of both treatments (2.47–2.62), with no differences between dietary treatments. Minor differences were established for the majority of carcass and pork characteristics between experimental groups. Carcass yields were approximately 75.0–76.0%. Males of both dietary groups had 7–8% higher (*p* = 0.011) loin subcutaneous fat thickness with values around 17–18 mm, whereas females had values of around 16–16.5 mm. Both sexes fed the low-CP diet had higher fat content than control animals of both sexes with respectively with results over 4% in the latter and 3–3.5% in the former. Similar differences were found for saturated fat contents, with control animals showing values between 0.81–1.00% and low CP animals having results of 1.34%. No differences were recorded for carcass pH, thawing losses, color traits, and protein content, as well as sensory traits (hardness, cohesiveness, and springiness).

### 3.2. Differential Proteomics Analysis

**Figure 1:**
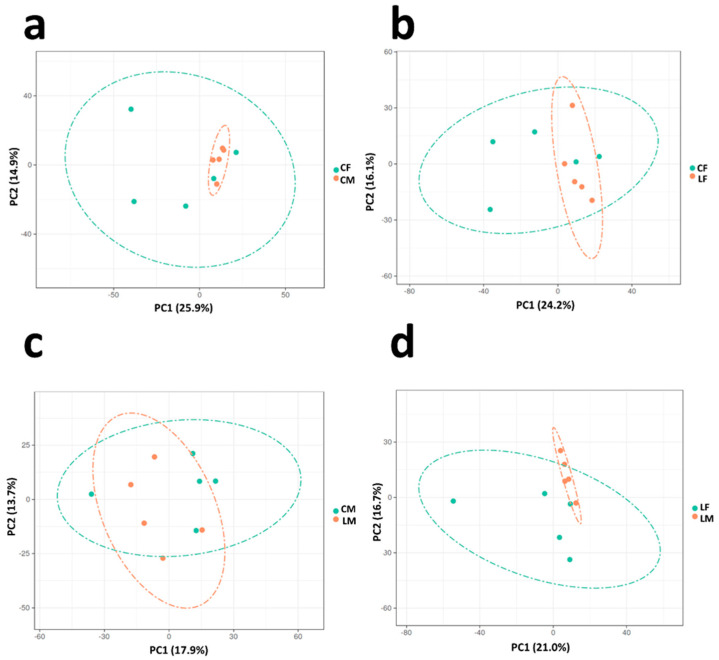
Principal Component Analysis (PCA) for each comparison: (**a**) CF—green- vs. CM—orange-; (**b**) CF—green- vs. LF—orange-. (**c**) CM—green- vs. LM—orange-; and (**d**) LF—green- vs. LM—orange-. CM—Control Males, CF—Control Females, LM—Low Crude Protein Diet Males, and LF—Low Crude Protein Diet Females.

Proteomics analysis allowed the identification of over 3000 proteins in total. Of these, 1922 were considered for differential expression analysis according to the criteria previously described. We identified the following number of differentially abundant proteins: Control females vs. Control males: 26 proteins; Low CP females vs. Control Females: 19 proteins; Low CP males vs. Control males: 12 proteins; and Low CP females vs. Low CP males: 11 proteins. The differentially accumulated protein list per comparison is displayed in Table 1, Table 2, Table 3 and Table 4, which are detailed after each of the four comparison headings Detailed results for all proteins and comparisons are presented in Supplementary Material S2. The PCA (Principal Component Analysis) for each comparison is displayed in Figure 1. Differences between the four comparisons considered in this study are addressed in the following sections.

#### 3.2.1. Control Male vs. Control Female Comparison

Proteins showing differential accumulation (DAPs) between Control Males and Control Females are displayed in Table 1. A clear clustering was noticeable between sexes when fed no limiting CP diets (Figure 1a). In total, 26 proteins were found with differential expression, three in males and 23 in females. Major biological processes vary and include RNA metabolism (2 DAPs), Cell organization and biogenesis (8 DAPs), Developmental Processes (2 DAPs), Cell Cycle (1 DAP), Transport (2 DAPs), Protein Metabolism (1 DAP), Signal Transduction (1 DAP), and Other Biological/Metabolic Processes (7 DAPs).

**Table 1:** Differentially Accumulated Proteins in the Control Male vs. Control Female comparison.

Accession Number	Protein Name	Unique Peptides	Abundance Ratio: CF/CM	Adjusted *p* Value: CF/CM	Gene	Main Biological Process
F1RVS9	Peptidase inhibitor 16	2	0.347	0.001013751	PI16	Other biological processes
A0A5G2R6C5	DNA-directed RNA polymerases I, II, and III subunit RPABC3	2	0.416	1.36756 × 10^6^	POLR2H	RNA metabolism
A0A5G2QW66	Isochorismatase domain containing 2	2	0.433	0.012840564	ISOC2	Other biological processes
A0A287B510	Ras homolog family member G	2	2.011	0.003132865	RHOG	Cell organization and biogenesis
A0A0H5ANC0	Mimecan	14	2.017	0.00139652	OGN	Developmental processes
I3LCW1	Fatty acid synthase	62	2.069	0.001338133	FASN	Other metabolic processes
A0A5G2R2Y0	Microfibril-associated protein	3	2.077	0.004970294	MFAP5	Cell organization and biogenesis
A4GR69	Telethonin	16	2.080	0.001519769	TCAP	
A0A481D3P3	Transmembrane protein 43	3	2.113	0.029580554	TMEM43	Cell organization and biogenesis
F1S6B5	Fibromodulin	10	2.220	0.000363034	FMOD	Cell organization and biogenesis
I3LNY6	Nestin	33	2.230	0.000224964	NES	Cell cycle
A0A287ADH9	Chloride intracellular channel protein	5	2.263	0.028770902	CLIC4	Transport
A0A286ZXM0	PDZ domain-containing protein	2	2.394	0.015442094	AHNAK2	Other biological processes
F1S6B4	Prolargin	16	2.403	7.71728 × 10^−6^	PRELP	Other metabolic processes
A0A4 × 1SL89	Haptoglobin	17	2.434	4.9501 × 10^−6^	HP	Protein metabolism
F1SFI6	Fetuin B	11	2.448	0.010355923	FETUB	Other biological processes
A0A4X1W2B1	Tropomyosin 3	5	2.503	0.000321367	TPM3	
Q06AA4	U1 small nuclear ribonucleoprotein A	3	2.505	0.030643582	SNRPA	RNA metabolism
A0A287BC27	Leucine-rich repeat flightless-interacting protein 2	4	2.554	0.002376756	LRRFIP2	Other biological processes
A0A4X1VZ89	Heterochromatin protein 1-binding protein 3	6	2.791	0.020612845	HP1BP3	Cell organization and biogenesis
A0A8D0S0Y6	Non-histone chromosomal protein HMG-17	4	2.861	0.000110911	HMGN2	No biological process assigned
A0A286ZLR2	Non-specific serine/threonine protein kinase	2	2.930	0.018827026	DCLK1	Cell organization and biogenesis
F1RQI0	Collagen type XII alpha 1 chain	60	3.034	1.58739 × 10^−8^	COL12A1	Developmental processes
I3L7P7	Copper transport protein ATOX1	4	3.280	6.98032 × 10^−5^	ATOX1	Transport
A0A287BQ93	Mitogen-activated protein kinase	3	3.600	0.001823497	MAPK12	Signal transduction
F1S3E0	Transmembrane emp24 domain-containing protein 9	3	5.391	2.6839 × 10^−11^	TMED9	Cell organization and biogenesis

CF—Control Females; CM—Control Males.

#### 3.2.2. Low CP Female vs. Control Female Comparison

DAPs between Control Females and Low CP Females are displayed in Table 2. No clear clustering was noticeable between the two female groups based on dietary CP content (Figure 1b). A total of 19 proteins were found with differential expression, 15 in control females, and 4 in Low CP females. Biological processes include: cell organization and biogenesis (7 DAPs), cell adhesion (1 DAP), protein metabolism (1 DAP), stress response (1 DAP), and other metabolic or other biological processes (5 DAPs).

**Table 2:** Differentially Accumulated Proteins: Low CP Female vs. Control Female comparison.

Accession Number	Protein Name	Unique Peptides	Abundance Ratio: LF/CF	Adjusted *p* Value: LF/CF	Gene	Main Biological Process
F1S3E0	Transmembrane emp24 domain-containing protein 9	3	0.159	2.30539 × 10^−16^	TMED9	Cell organization and biogenesis
A7WLH8	Small ubiquitin-related modifier 1	2	0.182	1.77915 × 10^−13^	SUMO1	Protein metabolism
F1SK17	Perilipin 1	8	0.313	2.39698 × 10^−6^	PLIN1	Other metabolic processes
P05207	cAMP-dependent protein kinase type II-alpha regulatory subunit	3	0.335	3.90284 × 10^−5^	PRKAR2A	Other biological processes
A0A287AIW3	Dynein light intermediate chain	7	0.377	4.59333 × 10^−5^	DYNC1LI1	Cell organization and biogenesis
P61013	Cardiac phospholamban	2	0.396	6.02922 × 10^−6^	PLN	Cell organization and biogenesis
A0A4X1UMD5	Tubulin polymerization-promoting protein family member 3	11	0.407	6.07621 × 10^−5^	TPPP3	Cell organization and biogenesis
F1S6B4	Prolargin	16	0.429	2.10256 × 10^−5^	PRELP	Other metabolic processes
F1SCC7	Serpin domain-containing protein	6	0.435	0.000105201	LOC396684	No biological process assigned
A0A287A0I8	Drebrin 1	6	0.437	0.033932864	DBN1	No biological process assigned
A0A5G2QKX2	Ankyrin repeat domain 2	16	0.446	2.48421 × 10^−5^	ANKRD2	Other biological processes
F1SN67	Fibrillin 1	47	0.475	0.000406089	FBN1	Cell adhesion
A0A287BAW0	NADH dehydrogenase [ubiquinone] 1 alpha subcomplex subunit 5	7	0.479	0.002752956	NDUFA5	Cell organization and biogenesis
A0A5G2R2Y0	Microfibril-associated protein 5	3	0.481	0.001660983	MFAP5	Cell organization and biogenesis
F1SP56	Nipsnap homolog 3B	8	0.491	0.001995836	NIPSNAP3B	No biological process assigned
A0A4X1SUH2	Glutathione peroxidase	7	2.137	0.007988995	GPX1	Stress response
A0A287AI92	Carbonic anhydrase	3	2.428	0.000601199	CA1	Other metabolic processes
K7GR43	NADH dehydrogenase [ubiquinone] 1 alpha subcomplex subunit 1	2	2.803	7.58379 × 10^−8^	NDUFA1	Cell organization and biogenesis
F1RVS9	Peptidase inhibitor 16	2	3.636	4.77915 × 10^−7^	PI16	Other biological processes

LF—Low Crude Protein Females; CF—Control Females.

#### 3.2.3. Low CP Males vs. Control Males Comparison

DAPs between Low CP Males vs. Control Males Comparison are displayed in Table 3. Again, no clear clustering could be detected between the two entire male groups based on CP content of diets (Figure 1c). Only 12 proteins were found with differential expression: three in control Males and nine in Low CP Males. Biological processes include: protein metabolism (1 DAP), cell adhesion (1 DAP), cell organization and biogenesis (1 DAP), stress response (1 DAP), other metabolic, and other biological processes (4 DAPs).

**Table 3:** Differentially Accumulated Proteins in the Low CP Male vs. Control Male comparison.

Accession Number	Protein Name	Unique Peptides	Abundance Ratio: LM/CM	Adjusted *p* Value: LM/CM	Gene	Main Biological Process
A0A480T8S4	Mitochondrial-processing peptidase subunit beta	4	0.410	0.01510042	PMPCB	Protein metabolism
A0A4X1VYD8	Protein tyrosine phosphatase 4A2	2	0.425	0.000313165	PTP4A2	No biological process assigned
O02668	Inter-alpha-trypsin inhibitor heavy chain H2	9	0.44	0.000589175	ITIH2	Other metabolic processes
P02769	Bovine serum albumin precursor	17	2.003	8.82799 × 10^−5^	ALB	Stress response
A0A8W4FDW8	Sperm-associated antigen 8	4	2.021	0.002867223	SPAG8	No role assigned
A0A5G2QPJ7	Fumarylacetoacetase	5	2.070	0.005354906	FAH	Other metabolic processes
F1SCC9	Serpin domain-containing protein	5	2.214	0.044908208	LOC106504545	
A0A4X1W9B8	Canopy FGF signaling regulator 2	2	2.274	0.040164779	CNPY2	Other biological processes
F1S9W8	Kinesin light chain	9	2.587	0.001471845	KLC1	Other biological processes
A0A4X1W2B1	Tropomyosin 3	5	3.083	8.33538 × 10^−11^	TPM3	No biological process assigned
A0A8W4FDQ1	Myelin protein P0	7	3.570	1.89787 × 10^−16^	MPZ	Cell adhesion
F1SAR5	Glutaredoxin 5	2	5.557	1.38727 × 10^−10^	GLRX5	Cell organization and biogenesis

LM—Low Crude Protein Males; CM—Control Males.

#### 3.2.4. Low CP Female vs. Low CP Male Comparison

DAPs between Low CP Females and Low CP Males are displayed in Table 4. A clear clustering was noticeable between the two sex groups based on dietary CP content (Figure 1d). Eleven proteins were found with differential expression in this comparison: eight in Low CP Male and three in Low CP females. Biological processes include: cell adhesion (2 DAPs), cell organization (1 DAP), RNA metabolism or transcription (1 DAP), cell organization and biogenesis (1 DAP), signal transduction (1 DAPs), other biological processes, and other metabolic processes (3 DAPs).

**Table 4:** Differentially Accumulated Proteins in the Low CP Female vs. Low CP Male comparison.

Accession Number	Protein Name	Unique Peptides	Abundance Ratio: LF/LM	Adjusted *p* Value: LF/LM	Gene	Main Biological Process
A0A287B4R8	Acyl-CoA synthetase short-chain family member 3, mitochondrial	2	0.095	1.81568E-16	ACSS3	Other metabolic processes
F2Z5V3	NHP2-like protein 1	3	0.234	5.71814 × 10^−7^	SNU13	RNA metabolism
A0A8W4FDQ1	Myelin protein P0	7	0.289	1.47771 × 10^−10^	MPZ	Cell adhesion
A0A287AGW0	Serpin domain-containing protein	6	0.308	2.53406 × 10^−11^	LOC106504547	No biological process assigned
A0A4X1W943	Myosin light chain 6B	14	0.446	4.29249 × 10^−5^	MYL6B	No biological process assigned
F1SIK9	Septin	9	0.456	0.000195907	SEPTIN7	Other biological processes
A0A4X1SVF9	Periostin	9	0.469	0.044217747	POSTN	Cell adhesion
A0A4X1UMQ6	Ribosomal protein L37a	4	0.472	0.044217747	RPL37A	Protein metabolism
A0A5G2QSD6	Golgi-associated plant pathogenesis-related protein 1	2	2.039	0.011790049	GLIPR2	Other biological processes
A0A480PRD9	Tudor domain-containing protein 3	2	2.163	0.007072509	TDRD3	Cell organization and biogenesis
A0A287BQ93	Mitogen-activated protein kinase	3	3.617	0.000236537	MAPK12	Signal transduction

LF—Low Crude Protein Females; LM—Low Crude Protein Males.

## 4. Discussion

Proteomics has been extensively used in the framework of animal science. Specifically concerning the scope of this study, a proteomics approach provides detailed insights into the physiological mechanisms underlying muscle changes inherent to different levels of protein and AA nutrition according to the sex of the animals. To the best of our knowledge, this is the first study that specifically addresses the topic in fast-growing finishing pigs slaughtered at around 6 months of age to avoid boar taint, since the majority of studies focuses on heavy castrated males subjected to two different levels of nutrition differing in the Crude Protein level and amino acid supplementation, specifically targeting differences between entire males and females. Following this rationale, this discussion is divided into four sections. The first concerns differences between sexes fed the control diet. The second concerns differences between females fed the two diets. The third concerns differences between males fed the same diets, and, finally, the fourth section concerns differences between the two sexes when fed the low-CP diets. Given the lack of pertinence to this study, differences between entire males and females fed different diets were not considered.

### 4.1. Control Male vs. Control Female Comparison

In this comparison, 27 proteins were found to have differential expressions. The majority had higher abundance in Control males. Protein Peptidase inhibitor 16 was found in a higher expression in the CM group by comparison to Control Females. This protein has been linked to stress response in the myocardium muscle, in turn associated with inflammation and cardiomyocyte growth inhibition [25,26]. The higher accumulation of this protein in control males may be linked to the numerically higher growth rates recorded in these animals [9] that, at this age, would have attained the slaughter weight. Higher growth rates are, in turn, associated with an over-expression of stress-related proteins [27], thus explaining our results. Interestingly, haptoglobin has long been defined as an acute-phase protein in different domestic animal species, acting as an antioxidant [28]. The increased accumulation in females contrasts with the previously mentioned theory for higher growth rates in entire males and, consequently, higher over-expression of stress-related proteins. Similarly, Copper transport protein ATOX1, a transcription factor that has also been related to inflammation [29], was also found to have higher expression in control females, in accordance with what was previously suggested for haptoglobin. These results are therefore contradictory, and a consistent trend for stress-related proteins is not apparent. This may be the consequence of a similar physiological status for both sexes. Nonetheless, further research must be conducted to elucidate these results.

DNA-directed RNA polymerases are proteins associated with nuclear gene transcription [30] and have been shown to have increased accumulation in certain cancer types characterized by a profuse tissue growth [31]. Although tissue growth in these animals will necessarily have a very different pattern when compared to cancerous tissue growth, the pattern for increased gene expression and tissue differentiation may show similar patterns. In accordance, isochorismatase domain containing 2 is a protein that has been associated with the inhibition of tumor-suppressing proteins, thus contributing to increased rates of cell growth and differentiation [32]. In our study, these proteins were increased in control males. It could be inferred that such results are putatively linked to the higher growth rates in males, which implies higher muscle tissue growth. The results for DNA-directed RNA polymerases and isochorismatase domain containing 2 are, however, not in accordance with those of several proteins that have similar roles and that were found in higher abundance in females. For instance, Ras homolog family member G knockdown has been associated with altered expression of cell cycle regulators [33]. In our experiment, this protein was found in higher abundance in the control female muscle. Similarly, mimecan is a member of the Keratan sulfate family of proteoglycans, which are leucine-rich proteoglycans [34]. These authors have related the overexpression of this protein to enhanced apoptosis and migration. Such findings are consistent with our results that showed higher expression in females, thus pointing to lower growth rates and cell proliferation in this sex. A similar rationale may be applied to Fibromodulin and Nestin, proteins that are involved in the regulation of muscle differentiation to myocytes [35,36,37], thus explaining the higher accumulation in females. The higher muscle differentiation rate proposed for control females contrasts, however, with the results obtained for Transmembrane protein 43 (associated with muscle myopathies) [38] and Prolargin (associated with muscle dystrophy) [39]. It must be stated, however, that the biological role for these proteins is still not well defined.

Fatty acid synthase is a protein associated with muscle lipid anabolism, specifically the *de novo* synthesis of saturated fatty acids [40]. It has been demonstrated to show higher expression in fattier pig breeds such as Duroc by comparison to lean breeds such as Pietrain [41] or in obese Lantang pigs [42]. Furthermore, its decreased expression has been associated with decreased total cholesterol and triglyceride contents [43] in finishing male pigs, whereas castration leads to an increased upregulation of this protein [44]. Our results show an increased abundance of fatty acid synthase in control females when compared to control males. Such results are unexpected as they contrast with the higher subcutaneous fat thickness of loin found in control males and a lack of significant differences for lean percentage between control animals of both sexes [9]. Nevertheless, it is noteworthy that in mice the plasma concentration of this protein has been shown to be strongly influenced by diet, more than by sex [45], and studies in small ruminants reveal contradictory effects of sex on fatty acid synthase expression in skeletal muscle [46,47]. As such, a strong sex effect is not to be excluded. Microfibril-associated protein 5 (MFAP5) influences microfibril function and modulates major extracellular-signaling pathways [48], being downregulated in adipogenesis [49]. The higher accumulation of this protein in females in our study is thus in line with the results we have obtained for numerically higher contents of fat and saturated fat found in control males. A similar rationale could be proposed for Mitogen-activated protein kinase. This is a protein associated with lipid metabolism found to have decreased accumulation along the finishing period in steers [50]. As such, higher accumulation of this protein in females could be inferred as this sex is still at an earlier stage of the adipogenesis pathway, when compared to males, as proposed earlier. Telethonin, a muscle assembly-regulating factor, is one of the titin-interacting Z-disk proteins involved in the regulation and development of normal sarcomeric structure, being involved in the regulation of porcine skeletal muscle development [51]. Such roles are in line with our results. Indeed, and given the higher cacass fat thickness in control males by comparison to females, it could be suggested that the former had already reached their maximal muscle growth and are now increasing their adipogenesis, whereas the latter was still growing, drawing a putative parallel also with puberty. Results for Tropomyosin 3 seem to concur with this theory. Indeed, this protein is known to be implicated in functional diversity of skeletal muscle fibers, a regulator of muscle contraction, and extensive functions in muscle development [52]. Similarly, non-histone chromosomal protein HMG-17, involved in gene transcription [53], collagen type XII alpha 1 chain, associated with muscle cell development and differentiation [54], and Transmembrane emp24 domain-containing protein 9, associated with intracellular protein transport and embryo tissue development [55], are in accordance with the higher expression in females than in entire males with no limiting dietary CP.

### 4.2. Low CP Female vs. Control Female Comparison

This comparison retrieved a total of 19 proteins showing differential expression. The majority showed higher accumulation in control females and have been found to play major roles in cell/tissue growth and differentiation. For instance, transmembrane emp24 domain-containing protein 9, addressed also in the previous comparison, is a protein involved in intracellular protein transport and embryo tissue development [55]. Another example, the small ubiquitin-related modifier 1 (SUMO1), plays a central role in cell proliferation [56]. Dynein light intermediate chain physiological role in mammals is not completely understood. Nonetheless, it has long been linked with mitosis via assembly, organization, and function of centrosomes and mitotic spindles [57]. Another protein, Tubulin polymerization-promoting protein family member 3, has been reported to be associated with the initiation and progression of colorectal cancer in humans, and its knockdown has been shown to inhibit cell proliferation and induce apoptosis [58]. Drebrin is a protein involved in cell elongation, alignment, and fusion into syncytial myofibers, thus playing a major role in myoblast differentiation [59]. To the best of our knowledge, no specific role has been specifically attributed to Ankyrin repeat domain 2. However, the Ankyrin repeat domain 1 has been upregulated in bulls undergoing myogenesis [60]. Microfibril-associated protein 5 is down-regulated in adipogenesis [49] and up-regulated in head and neck squamous cell carcinoma during tumor replication [61]. It has been demonstrated that the NIPSNAP3A gene encoding for Nipsnap homolog 3A regulates cellular homeostasis and modulates mitochondrial dynamics, and its knockdown inhibited cell proliferation and attenuated apoptosis [62]. The physiological role of Fibrillin-1 in muscle is not fully understood. However, N-glycosylated forms of this protein have been found to be in higher abundance in the *Longissimus thoracis* muscle of high growth rate cattle-yak hybrids by comparison to yaks [63]. Finally, Phospholamban is a protein involved in the modulation of sarcoplasmic reticulum, specifically being a key regulator of cardiac contractility and modulating the reticulum Ca2+ sequestration [64]. This pattern of results seems to indicate that cell replication and differentiation pathways are increased in control females by comparison to low-CP diet females. This was somehow unexpected, given the fact that no differences regarding growth rates or carcass and meat characteristics between these two groups, with the exception of fat and saturated fat percentages, higher in the low crude protein content females [9]. Nonetheless, based on this pattern of results, it could be suggested that the control females were still undergoing muscle accretion-related biochemical pathways, involving cellular replication and differentiation, whereas the low CP females were no longer undergoing such processes, but were instead undergoing a process that promoted adipogenesis.

Two proteins related to lipid metabolism were found in higher abundance in control females: Perilipin 1 and Protein cAMP-dependent protein kinase type II-alpha regulatory subunit. Perilipin 1 is a protein found in porcine muscle, specifically localized in the periphery of intramuscular adipocytes [65]. According to these authors, it is associated with higher intramuscular fat contents, while other authors have demonstrated that this protein is down-regulated when muscle lipid accumulation declines [66]. Protein cAMP-dependent protein kinase type II-alpha regulatory subunit is also involved in lipid metabolism, and it has been shown to have an increased expression in animals fed diets with the incorporation of olive oil by comparison to dietary inclusion of other dietary fats [67] and has been found to be down-regulated in porcine muscle under LPS (Lipopolysaccharide) inflammation-like challenge [68]. The reason for the increased abundance of these two proteins in the control females warrants further research. Nevertheless, it could be suggested that such accumulation profiles could be linked specifically to carcass fat composition itself, which could explain the changes in intramuscular fat and saturated fat percentages, approximately 35% higher in females fed the restricted CP diet with amino acid supplementation than in females fed the control diet [9]. This is unlikely inherent to the fatty acid profile of both feeds that are very similar [9] but instead to differences in starch contents that are higher in the low CP diet [69]. A lipidomic and fatty acid composition study would therefore be of the utmost interest in order to ascertain such differences.

Four proteins were found to have increased abundance in the reduced CP female group: Peptidase inhibitor 16, Glutathione peroxidase, Carbonic anhydrase, and NADH dehydrogenase [ubiquinone] 1 alpha subcomplex subunit 1. As previously stated, peptidase inhibitor 16 is a stress-regulated transcript in humans [25]. Glutathione peroxidases have long been defined as relevant cellular antioxidant enzymes and regulators of apoptosis found in the cytoplasm and mitochondria of mammalian cells [70]. Antioxidant enzymes, such as Glutathione peroxidase, are increased in pig breeds with a higher tendency to deposit fat, such as the Laiwu Pig, when compared to Large White [71]. Dietary supplementation of antioxidants like tea tree oil [72] or dietary grape seed proanthocyanidin [73] has been shown to increase the activity of the enzyme or the accumulation of this protein. In both rodent and pig models, different forms of amino acid supplementation, in particular, Sulphur amino acids, have been shown to increase glutathione peroxidase expression and alleviate the antioxidant system [74,75,76]. In broilers, a combination of dietary low CP contents and essential amino acid supplementation has led to increased liver glutathione peroxidase enzyme activity under heat stress [77]. The above statement is in accordance with the results herein obtained and clearly demonstrates the beneficial effect of dietary CP reduction combined with amino acid supplementation in increasing the alleviation of the antioxidant system. This is, in turn, linked to the previously stated decrease in pathways related to muscle accretion in Low CP females by comparison to control females and an increase in the percentages of fat and saturated fat in the former group, albeit with no measurable consequences for growth traits [9]. As detailed by Ismail [78], carbonic anhydrase is related to hepatic glucose production, being involved in relevant physiological biosynthetic reactions like gluconeogenesis or lipogenesis. Accordingly, studies by Zhong et al. [79] have demonstrated that, in pigs, increased intramuscular fat content induced by in-feed conjugated linoleic acid is accompanied by an increase in the abundance of carbonic anhydrase.

### 4.3. Low CP Males vs. Control Males Comparison

This comparison retrieved a total of 12 proteins showing differential accumulation. Of these, three were found in higher abundance in control males. Mitochondrial-processing peptidase subunit beta was found in higher abundance in control males. Mitochondrial-processing peptidases are highly conserved across species, being involved in proteolytic processing of both nuclearly and mitochondrially encoded precursor polypeptides targeted to the various subcompartments of the mitochondria [80]. Protein tyrosine phosphatase 4A2 (PTP4A2) is a protein that has been specifically implicated in tumor progression and associated tumor growth [81]. Similarly, Inter-alpha-trypsin inhibitor heavy chain H2 (ITIH2) has recently been implicated in motility and invasiveness of cancer cells via the hyaluronan network [82]. Hyaluronan plays essential roles in the organization of tissue architecture and the regulation of cellular functions, such as cell proliferation and migration, through interactions with cell-surface receptors and binding molecules [83]. Specific reasons for the increased accumulation of these proteins in Control males are difficult to ascertain, given the limited information available on them concerning muscle metabolism. Nevertheless, the increased accumulation of proteins involved in energy metabolism, specifically through mitochondrial metabolism and cellular growth, could suggest that the muscle of control males was still undergoing tissue growth by comparison to animals fed the low CP diet.

Nine proteins were found in higher abundance in Low CP Males. Serum albumin precursor (P02769), a key protein involved in albumin synthesis, was found in higher accumulation in low CP-fed animals. Albumin synthesis has been found to be increased in macronutrient-restricted pigs undergoing colitis [84]. Fumarylacetoacetase is the last enzyme in the tyrosine catabolic pathway [85] and has been found to have increased accumulation in hibernating mammals [86]. A different trend was, however, recorded for both canopy FGF-signaling regulator 2 (CNPY2) and kinesin light chain. The former is a protein involved in cellular replication [87]. Knockdown of this protein has been associated with the inhibition of cellular hyperproliferation, cell cycle arrest, migration, and phenotypic transformation [88]. The latter is a protein thought to be involved in myoblast differentiation [89]. Tropomyosin 3 is a cytoskeletal protein [90], and its increased abundance has been demonstrated in Chinese pig breeds with a tendency for low growth rates and muscle accretion and higher fat deposition [91,92]. As such, a role in amino acid catabolism and, hence, in reduced protein synthesis could be extrapolated to this protein, which could be interpreted as low CP males having a lower rate of protein anabolism.

### 4.4. Low CP Female vs. Low CP Male Comparison

This comparison led to a total of 11 proteins showing differential accumulation. Three were found to have increased abundance in Low CP females. These three proteins have similar roles. Tudor domain-containing protein 3 (TDRD3) mediates transcriptional activation in nucleus and formation of stress granules in the cytoplasm, being implicated in cell proliferation and invasion in different types of cancer [93]. Similarly, mitogen-activated protein kinase (MAPK12) has been associated with cancer stem-like cell stimulation in breast cancer [94]. Finally, it has been demonstrated that Golgi-associated plant pathogenesis-related protein 1 (GLIPR2) is associated with the regulation of autophagy [95].

Eight proteins were found with higher abundance in Low CP males. The pattern of results is quite complex. Acyl-CoA synthetase short chain family member 3 (ACSS3) is a protein that represses prostate cancer progression [96], decreases fatty acid oxidation, enhances anaerobic glycolysis, increases Reactive Oxygen Species production, suppresses mitophagy, and promotes apoptosis [97]. Myosin light chain 6B (MYL6B) has been associated with the formation of pale, soft, and exudative (PSE) pork [98] and has been associated with the negative regulation of myoblast differentiation in pig muscle [99]. These results for these two proteins suggest that in Low CP males, and by comparison to Low CP females, proteins involved in cellular growth are being repressed, which could, in turn, be interpreted as the AA pool in the diet was more limiting to males than to females. Nonetheless, it is noteworthy that these results are in clear contrast with the results obtained for Septin, Periostin, and Large ribosomal subunit protein eL43. Septin, encoded by the SEPTIN7 gene, is associated with cell division, being essential for myotube differentiation [100] and muscle regeneration [101]. Accordingly, its repression has been shown to hinder skeletal muscle regeneration [102]. Periostin is an important regulator of bone, cardiac development, and wound healing, playing an important role in tumor development and being upregulated in different cancers [103]. According to these authors, in skeletal muscle, this gene is expressed similarly in both lean and fat swine genotypes, although results by other researchers point to increased adipogenesis when this gene is repressed [104].

## 5. Conclusions

**Figure 2:**
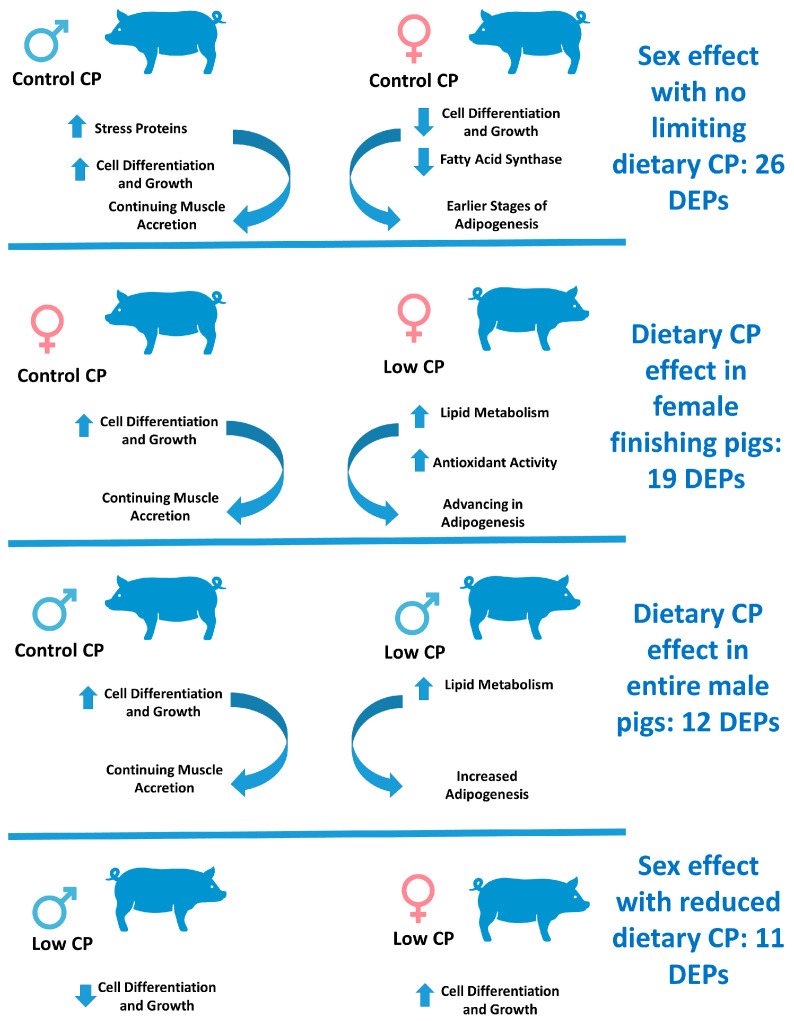
Schematic illustration of the major events underlying the proteome changes in entire male and female fast-growing finishing pigs subjected to a conventional diet with adequate Crude Protein content and a diet with a 2% Crude Protein content decrease supplemented with synthetic amino acids under the ideal protein concept. Upwards-pointing arrows denote increase while arrows downwards-pointing arrows denote decrease in physiological pathways.

We have conducted a trial with animals of both sexes subjected to control diets ideally formulated to have adequate CP content and reduced (2% reduction) CP diets with essential amino acid supplementation under the ideal protein concept. Herein, and on a physiological approach based on label-free proteomics, a summary of the major results is highlighted in Figure 2. Results from the Control animals of both sexes have several implications. On one hand, stress proteins were found in higher abundance in males and others in females. Similarly, proteins implicated in cellular growth and differentiation have also been shown to have higher abundances in both sexes. Fatty acid synthase, a protein involved in lipid anabolism, is increased in females. Overall, and despite the different trends recorded for this comparison, results indicate that males will have likely reach their maximal muscle growth and have now an increased development of the adipose tissue characterized by fat deposition. Females are, therefore, still growing and would likely increase their adipogenesis at a later stage. This is consistent with the productive results shown in our companion paper. Results obtained in the females fed the two diets demonstrate two divergent reactions. Indeed, control females were still undergoing a muscle accretion pattern that, from a proteomics analysis, was essentially characterized by the increased abundance of proteins related to cellular growth and differentiation. On the contrary, the Crude Protein dietary decrease leads to a protein expression profile where muscle accretion is decreased and fat growth is, in turn, stimulated. Finally, it is noteworthy to highlight that the decreased crude protein content and the amino acid supplemented group, either combined with the lipogenesis increase or to the latter, *per se*, led to relevant benefits to the antioxidant system. Overall, the comparison of the two entire male groups indicates that the majority of the proteins show higher accumulation in the muscle of Low CP males, leading to a trend towards decreased muscular growth and increased adipogenesis in the animals subjected to a lower CP diet and despite amino acid supplementation. On the contrary, it can be inferred that animals fed the control diet still showed a pattern towards the accumulation of proteins involved in tissue differentiation and, thus, in muscle accretion. This pattern is similar to the one described for females and likely suggests that a similar physiological response is obtained for both sexes. Taken together, results for the two sexes under the low CP diet indicate that females’ muscles were undergoing higher rates of cell replication and tissue growth, with extensive changes in their proteome when compared to males. Results from this study point to responses that comprise proteins involved in cellular replication that can be associated with muscle accretion, and, to some extent, proteins involved in adipogenesis and the development of the adipose tissue. As such, it is of the utmost importance to consider additional studies that would focus on both muscle and adipose tissue. A proposed approach could be the use of lipidomics tools, fatty acid profiling, in both muscle and adipose tissue. Specifically, regarding muscle samples used in this study, an additional approach involving metabolomics tools could also be suggested.

## Data Availability

Data are contained within the article or Supplementary Materials.

## Supplementary Materials

The following supporting information can be downloaded at: https://www.mdpi.com/article/10.3390/ani15223325/s1.

## References

[1] de Almeida A.M., Latorre M.A., Alvarez-Rodriguez J. (2024). Productive, Physiological, and Environmental Implications of Reducing Crude Protein Content in Swine Diets: A Review. Animals.

[2] Upadhaya S.D., Lee S.S., Kim Y.H., Wu Z., Kim I.H. (2022). Glutamic acid supplementation recovers the reduced performance of weanling pigs fed reduced crude protein diets. Anim. Nutr..

[3] Vonderohe C.E., Mills K.M., Liu S., Asmus M.D., Otto-Tice E.R., Richert B.T., Ni J.Q., Radcliffe J.S. (2022). The effect of reduced CP, synthetic amino acid supplemented diets on growth performance and nutrient excretion in wean to finish swine. J. Anim. Sci..

[4] Song W., Wu Z., Li W., Li Y. (2023). Multiple amino acid supplementations to reduce dietary protein for pigs during early and late finisher periods under commercial conditions. J. Sci. Food Agric..

[5] Dourmad J.Y., Jondreville C. (2007). Impact of nutrition on nitrogen, phosphorus, Cu and Zn in pig manure, and on emissions of ammonia and odours. Liv. Sci..

[6] Fuentes V., Ventanas S., Ventanas J., Estévez M. (2014). The genetic background affects composition, oxidative stability and qual-ity traits of Iberian dry-cured hams: Purebred Iberian versus reciprocal Iberian × Duroc crossbred pigs. Meat Sci..

[7] Cilla I., Altarriba J., Guerrero L., Gispert M., Martínez L., Moreno C., Beltrán J.A., Guàrdia M.D., Diestre A., Arnau J. (2006). Effect of different Duroc line sires on carcass composition, meat quality and dry-cured ham acceptability. Meat Sci..

[8] FEDNA Normas FEDNA—Necesidades Nutricionales para Ganado Porcino. https://www.fundacionfedna.org/sites/default/files/Normas%20PORCINO_2013rev2_0.pdf.

[9] de Almeida A.M., Latorre M.A., Ripoll G., Verdú M., Alvarez-Rodriguez J. (2024). Reducing Dietary Protein Levels with Amino Acid Supplementation in Duroc Sire Line Finishing Pigs: Growth Performances, Carcass and Meat Traits and Nitrogen Balance in Males and Females. Animals.

[10] Almeida A.M., Bassols A., Bendixen E., Bhide M., Ceciliani F., Cristobal S., Eckersall P.D., Hollung K., Lisacek F., Mazzucchelli G. (2015). Animal board invited review: Advances in proteomics for animal and food sciences. Animal.

[11] Almeida A.M., Ali S.A., Ceciliani F., Eckersall P.D., Hernández-Castellano L.E., Han R., Hodnik J.J., Jaswal S., Lippolis J.D., McLaughlin M. (2021). Domestic animal proteomics in the 21st century: A global retrospective and viewpoint analysis. J. Proteom..

[12] Paredi G., Raboni S., Bendixen E., de Almeida A.M., Mozzarelli A. (2012). “Muscle to meat” molecular events and technological transformations: The proteomics insight. J. Proteom..

[13] Paredi G., Sentandreu M.A., Mozzarelli A., Fadda S., Hollung K., de Almeida A.M. (2013). Muscle and meat: New horizons and applications for proteomics on a farm to fork perspective. J. Proteom..

[14] Sierra V., González-Blanco L., Diñeiro Y., Díaz F., García-Espina M.J., Coto-Montes A., Gagaoua M., Oliván M. (2021). New Insights on the Impact of Cattle Handling on Post-Mortem Myofibrillar Muscle Proteome and Meat Tenderization. Foods.

[15] Ribeiro D.M., Planchon S., Leclercq C.C., Raundrup K., Alves S.P., Bessa R.J.B., Renaut J., Almeida A.M. (2019). The muscular, hepatic and adipose tissues proteomes in muskox (*Ovibos moschatus*): Differences between males and females. J. Proteom..

[16] Ferreira A.M., Grossmann J., Fortes C., Kilminster T., Scanlon T., Milton J., Greeff J., Oldham C., Nanni P., Almeida A.M. (2017). The sheep (*Ovis aries*) muscle proteome: Decoding the mechanisms of tolerance to Seasonal Weight Loss using label-free proteomics. J. Proteom..

[17] Ribeiro D.M., Planchon S., Leclercq C.C., Dentinho M.T.P., Bessa R.J.B., Santos-Silva J., Paulos K., Jerónimo E., Renaut J., Almeida A.M. (2020). The effects of improving low dietary protein utilization on the proteome of lamb tissues. J. Proteom..

[18] Soares R., Franco C., Pires E., Ventosa M., Palhinhas R., Koci K., Martinho de Almeida A., Varela Coelho A. (2012). Mass spectrometry and animal science: Protein identification strategies and particularities of farm animal species. J. Proteom..

[19] de Almeida A.M., Bendixen E. (2012). Pig proteomics: A review of a species in the crossroad between biomedical and food sciences. J. Proteom..

[20] Ribeiro D.M., Coelho D., Costa M., Carvalho D.F.P., Leclercq C.C., Renaut J., Freire J.P.B., Almeida A.M., Mestre Prates J.A. (2024). Integrated transcriptomics and proteomics analysis reveals muscle metabolism effects of dietary *Ulva lactuca* and ulvan lyase supplementation in weaned piglets. Sci. Rep..

[21] Ribeiro D.M., Martins C.F., Kuleš J., Horvatić A., Guillemin N., Freire J.P.B., Eckersall P.D., Almeida A.M., Prates J.A.M. (2021). Influence of dietary Spirulina inclusion and lysozyme supplementation on the longissimus lumborum muscle proteome of newly weaned piglets. J. Proteom..

[22] Osório H., Silva C., Ferreira M., Gullo I., Máximo V., Barros R., Mendonça F., Oliveira C., Carneiro F. (2021). Proteomics Analysis of Gastric Cancer Patients with Diabetes Mellitus. J. Clin. Med..

[23] Hughes C.S., Moggridge S., Müller T., Sorensen P.H., Morin G.B., Krijgsveld J. (2019). Single-pot, solid-phase-enhanced sample preparation for proteomics experiments. Nat. Protoc..

[24] Sacarrão-Birrento L., Ribeiro D.M., Dittmann A., Alves S.P., Kunz L., Silva S., Venâncio C.A., de Almeida A.M. (2024). The effect of the production system on the proteomics profiles of the *Longissimus thoracis* muscle in *Arouquesa* cattle. J. Proteom..

[25] Hazell G.G., Peachey A.M., Teasdale J.E., Sala-Newby G.B., Angelini G.D., Newby A.C., White S.J. (2016). PI16 is a shear stress and inflammation-regulated inhibitor of MMP2. Sci. Rep..

[26] Regn M., Laggerbauer B., Jentzsch C., Ramanujam D., Ahles A., Sichler S., Calzada-Wack J., Koenen R.R., Braun A., Nieswandt B. (2016). Peptidase inhibitor 16 is a membrane-tethered regulator of chemerin processing in the myocardium. J. Mol. Cell. Cardiol..

[27] Kim N.K., Park H.R., Lee H.C., Yoon D., Son E.S., Kim Y.S., Kim S.R., Kim O.H., Lee C.S. (2010). Comparative studies of skeletal muscle proteome and transcriptome profilings between pig breeds. Mamm. Genome.

[28] Saco Y., Bassols A. (2023). Acute phase proteins in cattle and swine: A review. Vet. Clin. Pathol..

[29] Chen G.F., Sudhahar V., Youn S.W., Das A., Cho J., Kamiya T., Urao N., McKinney R.D., Surenkhuu B., Hamakubo T. (2015). Copper Transport Protein Antioxidant-1 Promotes Inflammatory Neovascularization via Chaperone and Transcription Factor Function. Sci. Rep..

[30] Girbig M., Misiaszek A.D., Müller C.W. (2022). Structural insights into nuclear transcription by eukaryotic DNA-dependent RNA polymerases. Nat. Rev. Mol. Cell. Biol..

[31] AZhaTi B., Wu G., Zhan H., Liang W., Song Z., Lu L., Xie Q. (2022). Alternative splicing patterns reveal prognostic indicator in muscle-invasive bladder cancer. World J. Surg. Oncol..

[32] Huang X., Shi Z., Wang W., Bai J., Chen Z., Xu J., Zhang D., Fu S. (2007). Identification and characterization of a novel protein ISOC2 that interacts with p16INK4a. Biochem. Biophys. Res. Commun..

[33] Jun J.H., Son M.J., Lee H.G., Shim K.Y., Baek W.K., Kim J.Y., Joo C.K. (2020). Regulation of Ras homolog family member G by microRNA-124 regulates proliferation and migration of human retinal pigment epithelial cells. Sci. Rep..

[34] Zhang H.J., Wang J., Liu H.F., Zhang X.N., Zhan M., Chen F.L. (2015). Overexpression of mimecan in human aortic smooth muscle cells inhibits cell proliferation and enhances apoptosis and migration. Exp. Ther. Med..

[35] Yin H., Cui C., Han S., Chen Y., Zhao J., He H., Li D., Zhu Q. (2020). Fibromodulin Modulates Chicken Skeletal Muscle Development via the Transforming Growth Factor-beta Signaling Pathway. Animals.

[36] Jan A.T., Lee E.J., Choi I. (2016). Fibromodulin: A regulatory molecule maintaining cellular architecture for normal cellular function. Int. J. Biochem. Cell. Biol..

[37] Lindqvist J., Torvaldson E., Gullmets J., Karvonen H., Nagy A., Taimen P., Eriksson J.E. (2017). Nestin contributes to skeletal muscle homeostasis and regeneration. J. Cell. Sci..

[38] Orgil B.O., Spaulding M.S., Smith H.P., Baba Z., Alberson N.R., Batsaikhan E., Towbin J.A., Purevjav E. (2025). Transmembrane Protein 43: Molecular and Pathogenetic Implications in Arrhythmogenic Cardiomyopathy and Various Other Diseases. Int. J. Mol. Sci..

[39] Ohlendieck K., Swandulla D. (2017). Molecular pathogenesis of Duchenne muscular dystrophy-related fibrosis. Pathologe.

[40] Renaville B., Bacciu N., Lanzoni M., Mossa F., Piasentier E. (2018). Association of single nucleotide polymorphisms in fat metabolism candidate genes with fatty acid profiles of muscle and subcutaneous fat in heavy pigs. Meat Sci..

[41] Reiter S.S., Halsey C.H., Stronach B.M., Bartosh J.L., Owsley W.F., Bergen W.G. (2007). Lipid metabolism related gene-expression profiling in liver, skeletal muscle and adipose tissue in crossbred Duroc and Pietrain Pigs. Comp. Biochem. Physiol. Part D Genom. Proteom..

[42] Qiu Y.Q., Yang X.F., Ma X.Y., Xiong Y.X., Tian Z.M., Fan Q.L., Wang L., Jiang Z.Y. (2017). CIDE gene expression in adipose tissue, liver, and skeletal muscle from obese and lean pigs. J. Zhejiang Univ. Sci. B.

[43] Wang W., Xu M., Diao H., Long Q., Gan F., Mao Y. (2024). Effects of grape seed proanthocyanidin extract on cholesterol metabolism and antioxidant status in finishing pigs. Sci. Rep..

[44] Yao Y.C., Cai Z.W., Zhao C.J., Wu K.L., Wu C.X., Han W.P., Xu N.Y. (2011). Influence of castration-induced sex hormone deficiency on serum lipid levels and the genes expression in male pigs. Horm. Metab. Res..

[45] Ramdhave A.S., Ojha S., Nandave M. (2017). Energy intake correlates with the levels of fatty acid synthase and insulin-like growth factor-1 in male and female C57BL/6 mice. Am. J. Transl. Res..

[46] An Q., Zeng L., Wang W., Yang J., Meng J., Zhao Y., Song X. (2024). Identification of FASN Gene Polymorphisms, Expression and Their Relationship with Body Size Traits in Guizhou White Goat (*Capra hircus*) with Different Genders. Genes.

[47] Kashani A., Holman B.W., Nichols P.D., Malau-Aduli A.E. (2015). Effect of dietary supplementation with Spirulina on the expressions of AANAT, ADRB3, BTG2 and FASN genes in the subcutaneous adipose and Longissimus dorsi muscle tissues of purebred and crossbred Australian sheep. J. Anim. Sci. Technol..

[48] Han C., Leonardo T.R., Romana-Souza B., Shi J., Keiser S., Yuan H., Altakriti M., Ranzer M.J., Ferri-Borgogno S., Mok S.C. (2023). Microfibril-associated protein 5 and the regulation of skin scar formation. Sci. Rep..

[49] Zhang T., Li H., Sun S., Zhou W., Zhang T., Yu Y., Wang Q., Wang M. (2023). Microfibrillar-associated protein 5 suppresses adipogenesis by inhibiting essential coactivator of PPARγ. Sci. Rep..

[50] Romao J.M., He M.L., McAllister T.A., Guan L.L. (2014). Effect of age on bovine subcutaneous fat proteome: Molecular mechanisms of physiological variations during beef cattle growth. J. Anim. Sci..

[51] Faulkner G., Lanfranchi G., Valle G. (2001). Telethonin and other new proteins of the Z-disc of skeletal muscle. IUBMB Life.

[52] Lambert M.R., Gussoni E. (2023). Tropomyosin 3 (TPM3) function in skeletal muscle and in myopathy. Skelet. Muscle.

[53] Dorbic T., Wittig B. (1987). Chromatin from transcribed genes contains HMG17 only downstream from the starting point of transcription. EMBO J..

[54] Yang G., Dai R., Ma X., Huang C., Ma X., Li X., La Y., Dingkao R., Renqing J., Guo X. (2024). Proteomic Analysis Reveals the Effects of Different Dietary Protein Levels on Growth and Development of Jersey-Yak. Animals.

[55] Zhou L., Li H., Yao H., Dai X., Gao P., Cheng H. (2023). TMED family genes and their roles in human diseases. Int. J. Med. Sci..

[56] Lazzarato L., Bianchi L., Andolfo A., Granata A., Lombardi M., Sinelli M., Rolando B., Carini M., Corsini A., Fruttero R. (2023). Proteomics Studies Suggest That Nitric Oxide Donor Furoxans Inhibit In Vitro Vascular Smooth Muscle Cell Proliferation by Nitric Oxide-Independent Mechanisms. Molecules.

[57] Purohit A., Tynan S.H., Vallee R., Doxsey S.J. (1999). Direct interaction of pericentrin with cytoplasmic dynein light intermediate chain contributes to mitotic spindle organization. J. Cell Biol..

[58] Ye K., Li Y., Zhao W., Wu N., Liu N., Li R., Chen L., He M., Lu B., Wang X. (2017). Knockdown of Tubulin Polymerization Promoting Protein Family Member 3 inhibits cell proliferation and invasion in human colorectal cancer. J. Cancer.

[59] Krauss R.S. (2017). Regulation of Skeletal Myoblast Differentiation by Drebrin. Adv. Exp. Med. Biol..

[60] Zhang Q., Lee H.G., Han J.A., Kang S.K., Lee N.K., Baik M., Choi Y.J. (2012). Differentially expressed proteins associated with myogenesis and adipogenesis in skeletal muscle and adipose tissue between bulls and steers. Mol. Biol. Rep..

[61] Wang Y., Wang R., Li B., Huang Z., Zhao S., Chen S., Lan T., Ren S., Wu F., Tan J. (2023). Cancer-associated fibroblasts in the invasive tumour front promote the metastasis of oral squamous cell carcinoma through MFAP5 upregulation. Gene.

[62] Yan R., Chen L., Cai Z., Tang J., Zhu Y., Li Y., Wang X., Ruan Y., Han Q. (2025). NIPSNAP3A regulates cellular homeostasis by modulating mitochondrial dynamics. Gene.

[63] Chang X., Wang J., Harlina P.W., Geng F. (2023). Quantitative N-Glycoproteomic Analysis of Cattle-Yak and Yak *Longissimus thoracis*. J. Agric. Food Chem..

[64] Frank K., Kranias E.G. (2000). Phospholamban and cardiac contractility. Ann. Med..

[65] Gandolfi G., Mazzoni M., Zambonelli P., Lalatta-Costerbosa G., Tronca A., Russo V., Davoli R. (2011). Perilipin 1 and perilipin 2 protein localization and gene expression study in skeletal muscles of European cross-breed pigs with different intramuscular fat contents. Meat Sci..

[66] Wang Y., Liu X., Hou L., Wu W., Zhao S., Xiong Y. (2015). Fibroblast Growth Factor 21 Suppresses Adipogenesis in Pig Intramuscular Fat Cells. Int. J. Mol. Sci..

[67] Park J.C., Kim S.C., Lee S.D., Jang H.C., Kim N.K., Lee S.H., Jung H.J., Kim I.C., Seong H.H., Choi B.H. (2012). Effects of Dietary Fat Types on Growth Performance, Pork Quality, and Gene Expression in Growing-finishing Pigs. Asian-Australas. J. Anim. Sci..

[68] Guo J., Liu Z., Sun H., Huang Y., Albrecht E., Zhao R., Yang X. (2015). Lipopolysaccharide challenge significantly influences lipid metabolism and proteome of white adipose tissue in growing pigs. Lipids Health Dis..

[69] van Milgen J., Noblet J., Dubois S. (2001). Energetic efficiency of starch, protein and lipid utilization in growing pigs. J. Nutr..

[70] Brigelius-Flohé R., Maiorino M. (2013). Glutathione peroxidases. Biochim. Biophys. Acta.

[71] Chen W., Zhu H.L., Shi Y., Zhao M.M., Wang H., Zeng Y.Q. (2012). Comparative Analysis on Antioxidative Ability of Muscle between Laiwu Pig and Large White. Asian-Australas. J. Anim. Sci..

[72] Yang T., Feng F., Zhan K., Ma X., Jiang M., Datsomor O., Zhu X., Huo Y., Zhao G. (2022). Effect of the Tea Tree Oil on Growth Performance, Meat Quality, Serum Biochemical Indices, and Antioxidant Capacity in Finishing Pigs. Front. Vet. Sci..

[73] Xu M., Chen X., Huang Z., Chen D., Li M., He J., Chen H., Zheng P., Yu J., Luo Y. (2022). Effects of dietary grape seed proanthocyanidin extract supplementation on meat quality, muscle fiber characteristics and antioxidant capacity of finishing pigs. Food Chem..

[74] Hunter E.A., Grimble R.F. (1997). Dietary sulphur amino acid adequacy influences glutathione synthesis and glutathione-dependent enzymes during the inflammatory response to endotoxin and tumour necrosis factor-alpha in rats. Clin. Sci..

[75] Yin J., Ren W., Duan J., Wu L., Chen S., Li T., Yin Y., Wu G. (2014). Dietary arginine supplementation enhances intestinal expression of SLC7A7 and SLC7A1 and ameliorates growth depression in mycotoxin-challenged pigs. Amino Acids.

[76] Holanda D.M., Marcolla C.S., Guimarães S.E.F., Neves M.M., Hausman G.J., Duarte M.S., Abreu M.L.T., Saraiva A. (2019). Dietary L-arginine supplementation increased mammary gland vascularity of lactating sows. Animal.

[77] Wang Z., Shao D., Kang K., Wu S., Zhong G., Song Z., Shi S. (2022). Low protein with high amino acid diets improves the growth performance of yellow feather broilers by improving intestinal health under cyclic heat stress. J. Therm. Biol..

[78] Ismail I.S. (2018). The Role of Carbonic Anhydrase in Hepatic Glucose Production. Curr. Diabetes Rev..

[79] Zhong W., Jiang Z., Zheng C., Lin Y., Yang L., Zou S. (2011). Relationship between proteome changes of Longissimus muscle and intramuscular fat content in finishing pigs fed conjugated linoleic acid. Br. J. Nutr..

[80] Gakh O., Cavadini P., Isaya G. (2002). Mitochondrial processing peptidases. Biochim. Biophys. Acta.

[81] Gao Y., Zhang M., Zheng Z., He Y., Zhu Y., Cheng Q., Rong J., Weng H., Chen C., Xu Y. (2017). Over-expression of protein tyrosine phosphatase 4A2 correlates with tumor progression and poor prognosis in nasopharyngeal carcinoma. Oncotarget.

[82] Lee S., Park J., Cho S., Kim E.J., Oh S., Lee Y., Park S., Kang K., Shin D.H., Ko S.Y. (2025). Hyaluronan network remodeling by ZEB1 and ITIH2 enhances the motility and invasiveness of cancer cells. J. Clin. Investig..

[83] Kobayashi T., Chanmee T., Itano N. (2020). Hyaluronan: Metabolism and Function. Biomolecules.

[84] Mackenzie M.L., Warren M.R., Wykes L.J. (2003). Colitis increases albumin synthesis at the expense of muscle protein synthesis in macronutrient-restricted piglets. J. Nutr..

[85] Holme E., Lindstedt S. (1998). Tyrosinaemia type I and NTBC (2-(2-nitro-4-trifluoromethylbenzoyl)-1,3-cyclohexanedione). J. Inherit. Metab. Dis..

[86] Pan Y.H., Zhang Y., Cui J., Liu Y., McAllan B.M., Liao C.C., Zhang S. (2013). Adaptation of phenylalanine and tyrosine catabolic pathway to hibernation in bats. PLoS ONE.

[87] Kakehashi A., Suzuki S., Shiota M., Raymo N., Gi M., Tachibana T., Stefanov V., Wanibuchi H. (2021). Canopy Homolog 2 as a Novel Molecular Target in Hepatocarcinogenesis. Cancers.

[88] Sun H., Wang X., Ma L., Li X., Jin W., Yang Y. (2024). CNPY2 governs PDGF-BB-treated vascular smooth muscle cell proliferation, migration and phenotypic transformation via the Akt/mTOR/GSK-3beta signaling pathway. Exp. Ther. Med..

[89] Qu Z., Shi L., Wu Z., Lin P., Zhang G., Cong X., Zhao X., Ge H., Yan S., Jiang L. (2024). Kinesin light chain 1 stabilizes insulin receptor substrate 1 to regulate the IGF-1-AKT signaling pathway during myoblast differentiation. FASEB J..

[90] Ropka-Molik K., Zukowski K., Eckert R., Gurgul A., Piórkowska K., Oczkowicz M. (2014). Comprehensive analysis of the whole transcriptomes from two different pig breeds using RNA-Seq method. Anim. Genet..

[91] Wang Z., Shang P., Li Q., Wang L., Chamba Y., Zhang B., Zhang H., Wu C. (2017). iTRAQ-based proteomic analysis reveals key proteins affecting muscle growth and lipid deposition in pigs. Sci. Rep..

[92] Wang W., Ma C., Zhang Q., Jiang Y. (2024). TMT-labeled quantitative malonylome analysis on the longissimus dorsi muscle of Laiwu pigs reveals the role of ACOT7 in fat deposition. J. Proteom..

[93] Liu J., Zhang S., Liu M., Liu Y., Nshogoza G., Gao J., Ma R., Yang Y., Wu J., Zhang J. (2018). Structural plasticity of the TDRD3 Tudor domain probed by a fragment screening hit. FEBS J..

[94] Qi X., Yin N., Ma S., Lepp A., Tang J., Jing W., Johnson B., Dwinell M.B., Chitambar C.R., Chen G. (2015). p38γ MAPK Is a Therapeutic Target for Triple-Negative Breast Cancer by Stimulation of Cancer Stem-Like Cell Expansion. Stem Cells.

[95] Zhao Y., Zou Z., Sun D., Li Y., Sinha S.C., Yu L., Bennett L., Levine B. (2021). GLIPR2 is a negative regulator of autophagy and the BECN1-ATG14-containing phosphatidylinositol 3-kinase complex. Autophagy.

[96] Zhou L., Song Z., Hu J., Liu L., Hou Y., Zhang X., Yang X., Chen K. (2021). ACSS3 represses prostate cancer progression through downregulating lipid droplet-associated protein PLIN3. Theranostics.

[97] Wang L., Yuan H., Li W., Yan P., Zhao M., Li Z., Zhao H., Wang S., Wan R., Li Y. (2024). ACSS3 regulates the metabolic homeostasis of epithelial cells and alleviates pulmonary fibrosis. Biochim. Biophys. Acta Mol. Basis. Dis..

[98] Zequan X., Yonggang S., Heng X., Yaodong W., Xin M., Dan L., Li Z., Tingting D., Zirong W. (2022). Transcriptome-based analysis of early post-mortem formation of pale, soft, and exudative (PSE) pork. Meat Sci..

[99] Galindo C.L., Nguyen V.T., Hill B., Easterday E., Cleator J.H., Sawyer D.B. (2022). Neuregulin (NRG-1beta) Is Pro-Myogenic and Anti-Cachectic in Respiratory Muscles of Post-Myocardial Infarcted Swine. Biology.

[100] Gönczi M., Ráduly Z., Szabó L., Fodor J., Telek A., Dobrosi N., Balogh N., Szentesi P., Kis G., Antal M. (2022). Septin7 is indispensable for proper skeletal muscle architecture and function. eLife.

[101] Ráduly Z., Szabó L., Dienes B., Szentesi P., Bana Á.V., Hajdú T., Kókai E., Hegedűs C., Csernoch L., Gönczi M. (2023). Migration of Myogenic Cells Is Highly Influenced by Cytoskeletal Septin7. Cells.

[102] Szabó L., Telek A., Fodor J., Dobrosi N., Dócs K., Hegyi Z., Gönczi M., Csernoch L., Dienes B. (2023). Reduced Expression of Septin7 Hinders Skeletal Muscle Regeneration. Int. J. Mol. Sci..

[103] Xu D., Ma H.M., Xiao D.F. (2016). Molecular characterization, tissue expression profile, and single nucleotide polymorphism analysis of the periostin gene in swine. Genet. Mol. Res..

[104] Mo D., Yu K., Chen H., Chen L., Liu X., He Z., Cong P., Chen Y. (2017). Transcriptome Landscape of Porcine Intramuscular Adipocytes during Differentiation. J. Agric. Food Chem..

