# Supplementary material for: Effect of a Reduced-Protein Diet Supplemented with Essential Amino Acids on the Muscle Proteome of Female and Entire Male Finishing Pigs
Source: Animals (Basel). 2025 Nov 18;15(22):3325. doi: 10.3390/ani15223325 (PMC12649446; doi:10.3390/ani15223325)
Supplement: Supplementary file 1 [file animals-15-03325-s001.zip › animals-3965957-supplementary/Supplementary Material/Supplementary Material S1.pdf]

Supplementary Material S1 - Experimental Diets (ingredients, analyzed amino acid composition and calculated nutrients).

**Ingredients (%)**	**Control CP**	**Low-CP**
Maize	50.00	50.00
Barley 9.6% CP	7.31	16.37
Wheat 10.2% CP	13.00	8.73
Wheat middling	6.00	8.00
Soybean meal 44% CP	10.22	4.00
Sunflower 36% CP	7.00	5.25
Lard	2.00	2.00
Soybean oil + lecithin	0.70	0.73
Calcium carbonate	1.08	1.28
Monocalcium phosphate	0.25	0.30
Sodium chloride	0.50	0.54
Formic acid	0.10	0.10
Vitamin-mineral premix [1]	0.20	0.20
Mycotoxin adsorbent	0.10	0.10
Sodium bicarbonate	-	0.16
L-Lysine sulphate 70%	0.93	1.20
Methionine hydroxy analogue	0.17	0.25
L-Tryptophan	0.04	0.07
L-Threonine	0.23	0.33
L-Valine	0.10	0.21
L-Isoleucine	-	0.13
**Analyzed amino acids**		
Crude Protein (%)	14.30	12.20
Total Lys (%)	0.96	0.95
Total Met (%)	0.26	0.21
Total Thr (%)	0.74	0.73
Total Trp (%)	0.21	0.21
Total Val (%)	0.71	0.69
Total Ile (%)	0.58	0.55
Total Arg (%)	0.96	0.72
Total His (%)	0.40	0.32
Total Leu (%)	1.13	0.94
Total Phe (%)	0.70	0.57
**Calculated nutrients**		
DM (%)	86.9	87.0
Net energy for growing pigs (MJ/kg)	10.2	10.2
CP (%)	14.6	12.5
SID Lys (%)	0.87	0.87
SID Met (%)	0.24	0.19
SID Met+Cys (%)	0.47	0.39
SID Thr (%)	0.65	0.65
SID Trp (%)	0.18	0.19
SID Val (%)	0.62	0.61
SID Ile (%)	0.50	0.49
Starch (%)	45.7	48.3
Crude Fiber (%)	4.20	4.10
Ether extract (%)	4.8	4.80
g SID Lys/Mcal NE	3.55	3.55

[1]Vitamin, mineral and enzyme premix (per kg of complete diet): 6 IU Vitamin A; 1.5 IU Vitamin D3; 15 mg α-tocopherol; 1 mg Vitamin B2; 3 mg Vitamin B2; 1.5 mg Vitamin B6; 0.02 mg Vitamin B12; 0.1 mg folic acid; 20 mg nicotinic acid; 10 mg pantothenic acid; 0.1 biotin; 1 g choline chloride; 94 mg Zn (ZnO); 40 mg Mn (MnO);100 mg Fe (FeCO3); 19 mg Cu (CuSO4·5H2O); 0.34 mg Se (Na2O3Se); 50 mg BHT; 0.34 mg I (KI); 750 FYT 6-phytase (Quantum Blue, ABVista, UK; E. coli-sourced). DM—dry matter, CP—crude protein, SID—standard ileal digestibility, NE—net energy.
